# Analysis of circadian rhythm components in EEG/EMG data of aged mice

**DOI:** 10.3389/fnins.2023.1173537

**Published:** 2023-05-12

**Authors:** Kosaku Masuda, Yoko Katsuda, Yasutaka Niwa, Takeshi Sakurai, Arisa Hirano

**Affiliations:** ^1^Institute of Medicine, University of Tsukuba, Tsukuba, Ibaraki, Japan; ^2^International Institute for Integrative Sleep Medicine (WPI-IIIS), University of Tsukuba, Tsukuba, Ibaraki, Japan; ^3^Graduate School of Medicine, Hirosaki University, Hirosaki, Aomori, Japan

**Keywords:** sleep, aging, circadian rhythms, EEG, machine learning

## Abstract

Aging disrupts circadian clocks, as evidenced by a reduction in the amplitude of circadian rhythms. Because the circadian clock strongly influences sleep–wake behavior in mammals, age-related alterations in sleep–wake patterns may be attributable, at least partly, to functional changes in the circadian clock. However, the effect of aging on the circadian characteristics of sleep architecture has not been well assessed, as circadian behaviors are usually evaluated through long-term behavioral recording with wheel-running or infrared sensors. In this study, we examined age-related changes in circadian sleep–wake behavior using circadian components extracted from electroencephalography (EEG) and electromyography (EMG) data. EEG and EMG were recorded from 12 to 17-week-old and 78 to 83-week-old mice for 3 days under light/dark and constant dark conditions. We analyzed time-dependent changes in the duration of sleep. Rapid eye movement (REM) and non-REM (NREM) sleep significantly increased during the night phase in old mice, whereas no significant change was observed during the light phase. The circadian components were then extracted from the EEG data for each sleep–wake stage, revealing that the circadian rhythm in the power of delta waves during NREM sleep was attenuated and delayed in old mice. Furthermore, we used machine learning to evaluate the phase of the circadian rhythm, with EEG data serving as the input and the phase of the sleep–wake rhythm (environmental time) as the output. The results indicated that the output time for the old mice data tended to be delayed, specifically at night. These results indicate that the aging process significantly impacts the circadian rhythm in the EEG power spectrum despite the circadian rhythm in the amounts of sleep and wake attenuated but still remaining in old mice. Moreover, EEG/EMG analysis is useful not only for evaluating sleep–wake stages but also for circadian rhythms in the brain.

## 1. Introduction

Many biological processes exhibit circadian rhythms that help organisms adapt to environmental cycles. These rhythms are regulated by the circadian clock, which is altered with aging (Hood and Amir, 2017). In mice, a long free-running period of the behavioral rhythm and a decrease in the ability to entrain environmental cycles have been observed (Nakamura et al., 2011; Sellix et al., 2012). Aging also affects sleep–wake behavior in animals, including humans (Mander et al., 2017). In mice, the amount of sleep during the dark period generally increases, whereas wakefulness decreases with age (Wimmer et al., 2013; Panagiotou et al., 2017; McKillop et al., 2018). In addition to the sleep/wake ratio, the electroencephalography (EEG) power changes with age, suggesting that aging induces both quantitative and qualitative changes in sleep.

Sleep–wake patterns are strongly governed by the circadian clock, as evidenced by the considerable effect of mutations in the core clock genes on sleep–wake rhythms (Wisor et al., 2002; Laposky et al., 2005; Mang et al., 2016). Conversely, sleep deprivation and other sleep interventions also perturb circadian rhythms, as demonstrated by studies investigating the impact of such interventions on behavioral and physiological characteristics (Wisor et al., 2008; Hoekstra et al., 2021; Lu et al., 2021). These findings suggest a bidirectional relationship between sleep and the circadian clock, both of which have a profound mutual influence on each other. Nonetheless, sleep and circadian behavioral rhythms are commonly studied independently. Circadian behavior is often exemplified by the use of long-term activity recordings using wheels or infrared sensors under constant dark conditions (DD) to measure behavioral rhythms in rodents. Although such methods can capture free-running wheel-running/spontaneous activity rhythms or phase responses to stimulation, they present significant challenges in accurately tracking sleep–wake stages, such as wakefulness, rapid eye movement (REM) sleep, and non-REM (NREM) sleep, as well as in assessing qualitative changes in sleep.

In contrast, sleep evaluation typically involves EEG and electromyography (EMG). EEG represents brain activity patterns that are obtained from electrodes positioned near the surface of the mouse brain (dura mater) and is an important indicator for discerning sleep stages. However, analyzing EEG/EMG data is more time-consuming and complex than simply counting wheel-running activity sessions, which makes it challenging to use EEG/EMG to assess sleep for a long duration. Consequently, analysis evaluating circadian rhythms of sleep amount and quality using EEG/EMG has been relatively uncommon. Previous studies have evaluated diurnal variations in delta power during NREM sleep as an indicator of sleepiness, revealing periodic changes throughout the day (Wisor et al., 2008; Wimmer et al., 2013). Nevertheless, there have been limited comprehensive analyses of circadian fluctuations in the EEG power spectrum, and the impact of aging on these circadian components remains largely unknown.

In this study, we conducted a precise evaluation of circadian rhythms in sleep–wake patterns of aged mice, with a particular focus on the EEG power spectrum. We first confirmed that changes in sleep–wake patterns are associated with aging, as previously shown. We extracted circadian components from the EEG power spectrum of each sleep–wake stage and observed that time-dependent changes in the power spectrum during NREM sleep were significantly attenuated in aged mice. Furthermore, we developed a machine learning-based method to reconstruct circadian phases from short-term (1 h) EEG data, which enabled us to assess age-mediated circadian phase alterations at a higher time resolution. Specifically, we found that the circadian phase was significantly delayed at all sleep–wake stages in aged mice. Our method represents a promising high-throughput approach for evaluating the circadian phase and sleep scoring in future studies.

## 2. Materials and methods

### 2.1. Animals

All animal experiments were approved by the Animal Experiment and Use Committee of the University of Tsukuba and adhered to NIH guidelines. C57BL/6 J wild-type male mice, purchased from The Jackson Laboratory, were used for EEG measurements. The young (*n* = 13) and aged (*n* = 20) mice were 12–17 weeks and 78–83 weeks old, respectively. Food and water were provided *ad libitum*.

### 2.2. Surgery

Male mice were anesthetized by isoflurane inhalation (Pfizer, United States) and fixed to a stereotaxic frame. The electrodes were implanted on the mouse cortex. Two stainless-steel screws for EEG recording were placed, each at +1.4 mm AP and + 1.2 mm ML from the bregma and − 2.6 mm AP and + 1.2 mm ML from the lambda and connected to a 4-pin headmount. Two insulated silver wires for EMG recording, connected to the same headmount, were inserted into the neck muscles bilaterally. An anchor screw was then positioned on the skull. The entire assembly was fixed to the skull with dental cement. The mouse skin was sutured using a sanitized thread.

### 2.3. EEG/EMG recording

EEG/EMG was recorded from freely moving mice as described previously (Hasegawa et al., 2022). Mice were entrained to a 12:12 light–dark (LD) cycle with lights turning on at 8:00 am and off at 8:00 pm. They were single-housed in a recording chamber for at least 3 days to habituate to the environment. EEG and EMG were recorded for 3 days under the LD condition 1–3 weeks after the electrode implantation surgery. The EEG/EMG signals were amplified and filtered (EEG: 0.5–250 Hz, EMG: 16–250 Hz). 50 Hz EEG and EMG signals were also filtered since the power supply frequency in Japan (50 Hz) possibly causes artificial noise signals. The EEG and EMG signals were acquired using SleepSignRecorder (KISSEI COMTEC, Nagano, Japan) at a sampling rate of 128 Hz, which is the same frequency as used in the previous study for the neural network model described later (Tezuka et al., 2021). Eight young mice and 10 aged mice were kept in the LD condition for 4 days after the measurement and then transferred to DD. The EEG and EMG of these mice were measured again from the second day in DD for 3 days. Environmental time is presented as zeitgeber time (ZT) under both LD and DD conditions.

### 2.4. Automated sleep–wake stage scoring using a neural network model

Sleep–wake stages were determined based on the UTSN-L model, which is a neural network model for automatic sleep–wake stage scoring developed in a previous study (Tezuka et al., 2021) using EEG/EMG data for 3 days in LD or DD. This model is a convolutional neural network using a one-channel EEG (raw signal and spectrum) and zeitgeber time, and shows 90% overall accuracy. This model classifies every 10 s-EEG data into one of the following sleep–wake stages: wakefulness (WAKE), REM sleep, or NREM sleep. Because this model also uses past EEG data for prediction, it does not output results for the first 100 s. Therefore, the sleep–wake stages for the first 100 s were manually classified. The parameters for the model trained in a previous study^1^ were also used for neural network scoring in this study. Because this model uses only EEG data, wakefulness was determined again by the threshold of EMG power after scoring. First, the log_10_ value of the standard deviation of the EMG power every 10 s was calculated as the amplitude. The threshold value was determined by applying Otsu’s binarization (Otsu, 1979) to the amplitudes for all time periods. The epochs in which the EMG amplitude exceeded the threshold were determined as WAKE, regardless of the result of the neural network scoring. After sleep–wake stage scoring, the hourly averages of sleep/wake amounts were calculated, and the amplitude, phase, and period of the rhythm of each sleep–wake stage were determined by cosine fitting. Cosine fitting was performed with the least squares method using Python (lmfit).^2^

### 2.5. Calculation of circadian components in EEG power spectra

Fourier transform was performed for the EEG data every 10 s to obtain the EEG power spectra at each epoch, which corresponds to sleep–wake stage scoring. EEG power spectra were obtained for every 0.1 Hz from 0 to 16 Hz. The standard deviation of the EMG data every 10 s was calculated and defined as the EMG amplitude. The hourly average EEG power spectra and EMG amplitude for each sleep–wake stage were then calculated. Next, to determine the amplitude and phase of the EEG power rhythms, we obtained the amplitude *A* and the peak time *t_p_* of the EEG power at each frequency. The EEG power was normalized as follows:


(1) $$EEG_{norm,i,f}=\left(EEG_{hourly,i,f}-EEG_{ave,f}\right)/EEG_{ave,f}$$

(2) $$EEG_{ave,f}=\frac{1}{T}\overset{T}{\underset{i=1}{\sum }}EEG_{hourly,i,f}$$

where $EEG_{hourly,i,f}\;\mathrm{and}\;EEG_{norm,i,f}$ are the hourly averaged EEG power and normalized EEG power at the *i* time point at the frequency *f*, respectively, $EEG_{ave,f}$ is the average EEG power, and *T* is the measurement length (*T* = 72 h). This calculation was performed for all frequencies. The first Fourier component was then obtained using the following equation:


(3) $$\begin{array}{c} COS=\frac{2}{T}\overset{T}{\underset{i=1}{\sum }}EEG_{norm,i}\cos\left(2\pi \frac{i\Delta t}{\tau }\right),\\ SIN=\frac{2}{T}\overset{T}{\underset{i=1}{\sum }}EEG_{norm,i}\sin\left(2\pi \frac{i\Delta t}{\tau }\right), \end{array}$$

where *τ* = 24 h and the average interval Δt = 1 h. Using COS and SIN , the amplitudes A and peak time $t_{p}$ were determined as follows:


(4) $$A=\sqrt{COS^{2}+SIN^{2}},$$

(5) $$t_{p}=\arctan2\left(SIN,COS\right)/2\pi *24$$

We applied these calculations to EEG power rhythms at each frequency and sleep–wake stage.

### 2.6. Estimation of circadian time using EEG power spectra

We constructed phase estimation models using machine learning for the correspondence between the EEG power spectra of each sleep–wake stage and environmental time. The models were constructed using the normalized EEG power spectra of each sleep–wake stage as the input and the environmental time as the output. The spectral distribution was normalized by dividing each spectrum by the sum of the spectral densities. The environmental time is represented as a point on a circular unit, that is, {cos(ZT/24∗2π),sin(ZT/24∗2π)} , where *ZT* represents zeitgeber time, and the model was constructed for the *x* and *y* components. The subjective circadian time was estimated from the angle between *x* and *y*, that is, estimated time = arctan2(y,x)/2π∗24 . We used linear regression, the k-nearest neighbor method, a support vector machine, and a random forest algorithm as the regression models. Similarly, we estimated the subjective circadian time using the hourly averages of the spectrum without distinguishing sleep–wake stages and the spectral distribution of all sleep–wake stages as input. The Python library “scikitlearn” (version 1.2.0)^3^ was used to construct each model. Default parameters were used for all the hyperparameters of each regression model. In the random forest algorithm, the importance of each input for estimation was also calculated using this library.

We evaluated the accuracy of the models by leave-one-out cross-validation using the LD condition of young mice as a control. First, we selected one of the mice, trained the model using the EEG data of the other mice, and predicted the subjective time of the selected mouse based on the trained model. This process was repeated for all mice to verify the accuracy of the model. Here, the accuracy of the estimation model was defined as the mean value of cos{(Estimated time−ZT)/24∗2π} . When the estimation error is small, this value approaches 1, and when the estimated time is in reverse phase, i.e., Estimated time−ZT=12 , it approaches −1. Therefore, the closer this value is to 1, the more accurate the prediction. For mice other than young and in the LD condition, prediction models were constructed based on data from all young mice in the LD condition, and the subjective time was predicted based on that model.

## 3. Results

### 3.1. Sleep–wake patterns in young and aged mice

**Figure 1:**
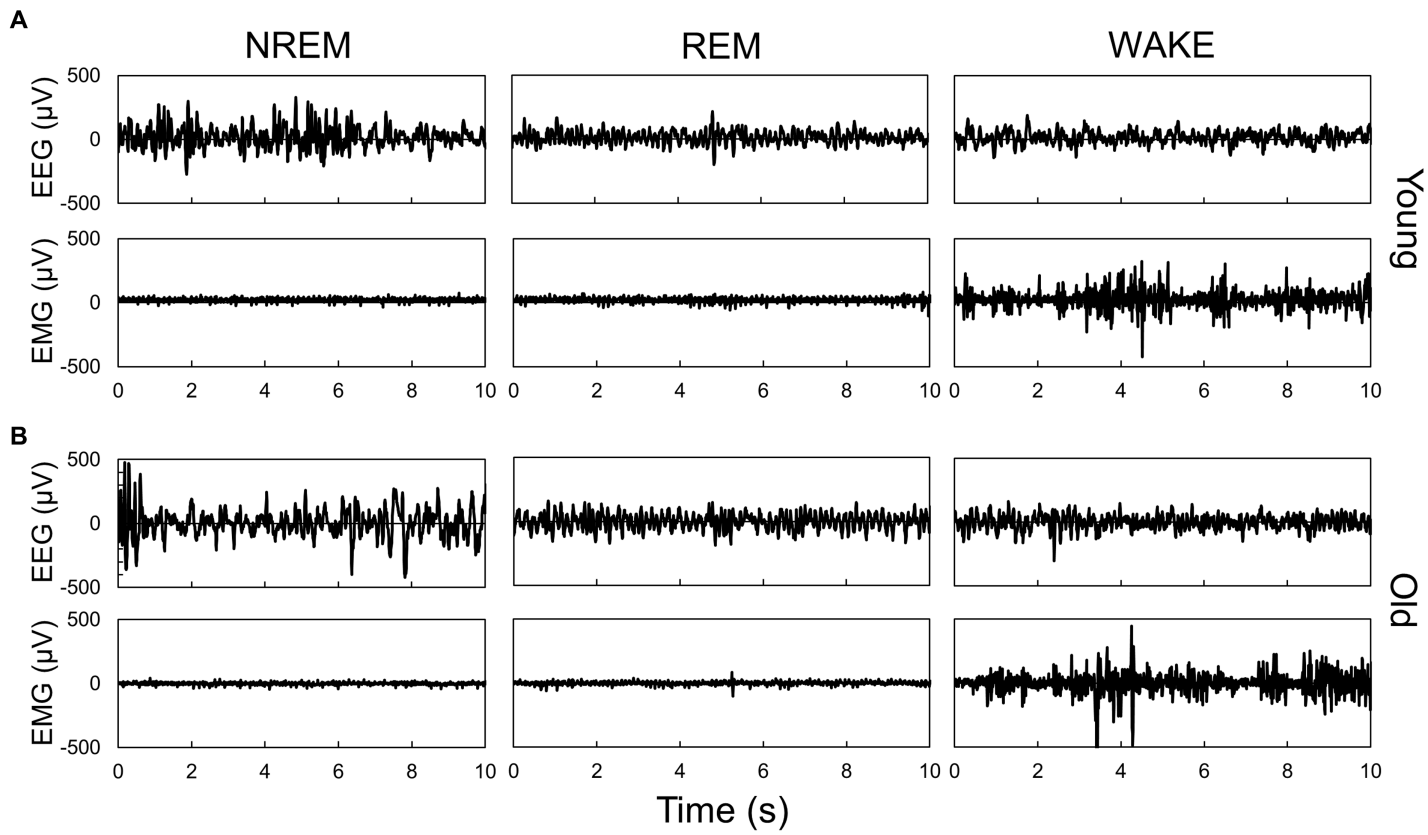
Representative EEG and EMG signals during each sleep–wake stage in young **(A)** and old **(B)** mice. Sleep–wake stage was determined by the neural network model. Each panel shows typical signals during each stage, which were scored by the neural network model.

**Figure 2:**
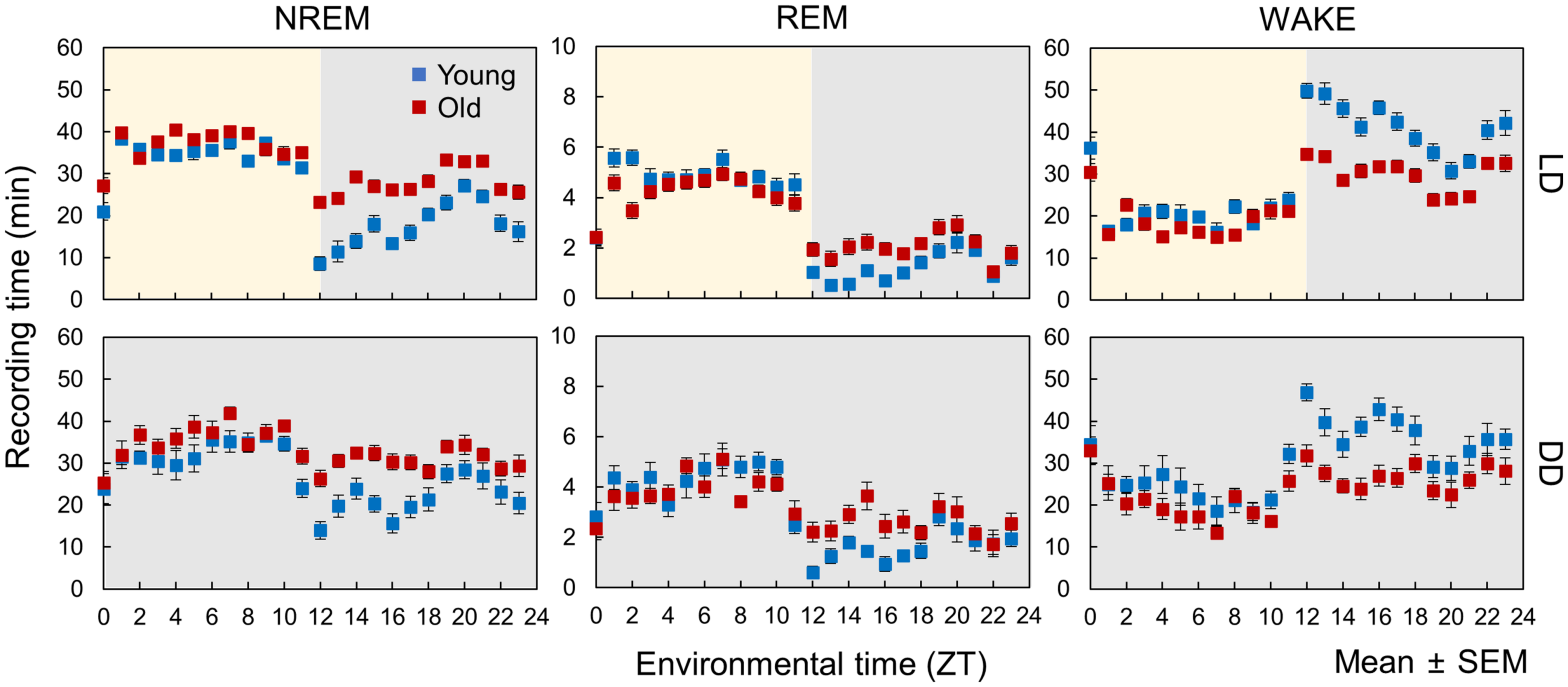
Sleep–wake patterns in young and old mice. Each point is the amount of each sleep–wake stage per hour (*n* = 13 in young mice and 20 in old mice in LD, 8 in young mice and 10 in old mice in DD conditions). Error bars indicate standard error. Blue and red dots represent values for young and old mice, respectively. Yellow and gray areas indicate light and dark conditions, respectively. The results of the comparison of young and old mice by *t*-test at each time point are shown in Supplementary Figure S2.

In this study, we employed a neural network model (Tezuka et al., 2021) to analyze the sleep–wake stages of young (12–17 weeks old) and old mice (78–83 weeks old) under LD and DD conditions (Figure 1). To evaluate the accuracy of the automated scoring, a portion of the EEG data was manually scored (Supplementary Figure S1). The results indicated that during the dark period (ZT12-24), the duration of NREM and REM sleep increased, and wakefulness time decreased in old mice, whereas small changes were observed during the light period under LD conditions (Figure 2; Supplementary Figure S2). Young mice showed a sharp increase in wakefulness after the light-to-dark transition (ZT12), whereas the change was more gradual in aged mice. These results are consistent with those of previous studies (Wimmer et al., 2013; Panagiotou et al., 2017; McKillop et al., 2018). Likewise, in old mice under DD, there was an increase in the amount of sleep and a decrease in wakefulness during the subjective night. The amplitude of the sleep–wake rhythm, as calculated by their amounts, was decreased, and the phase was slightly delayed in old mice when the phase was calculated using the amounts of NREM and REM sleep (Supplementary Figures S3, S4). Additionally, old mice showed a large distribution in the peak time of each stage, suggesting a variation in the degree of circadian phase disturbance among aged individuals.

### 3.2. Circadian rhythm components in EEG

**Figure 3:**
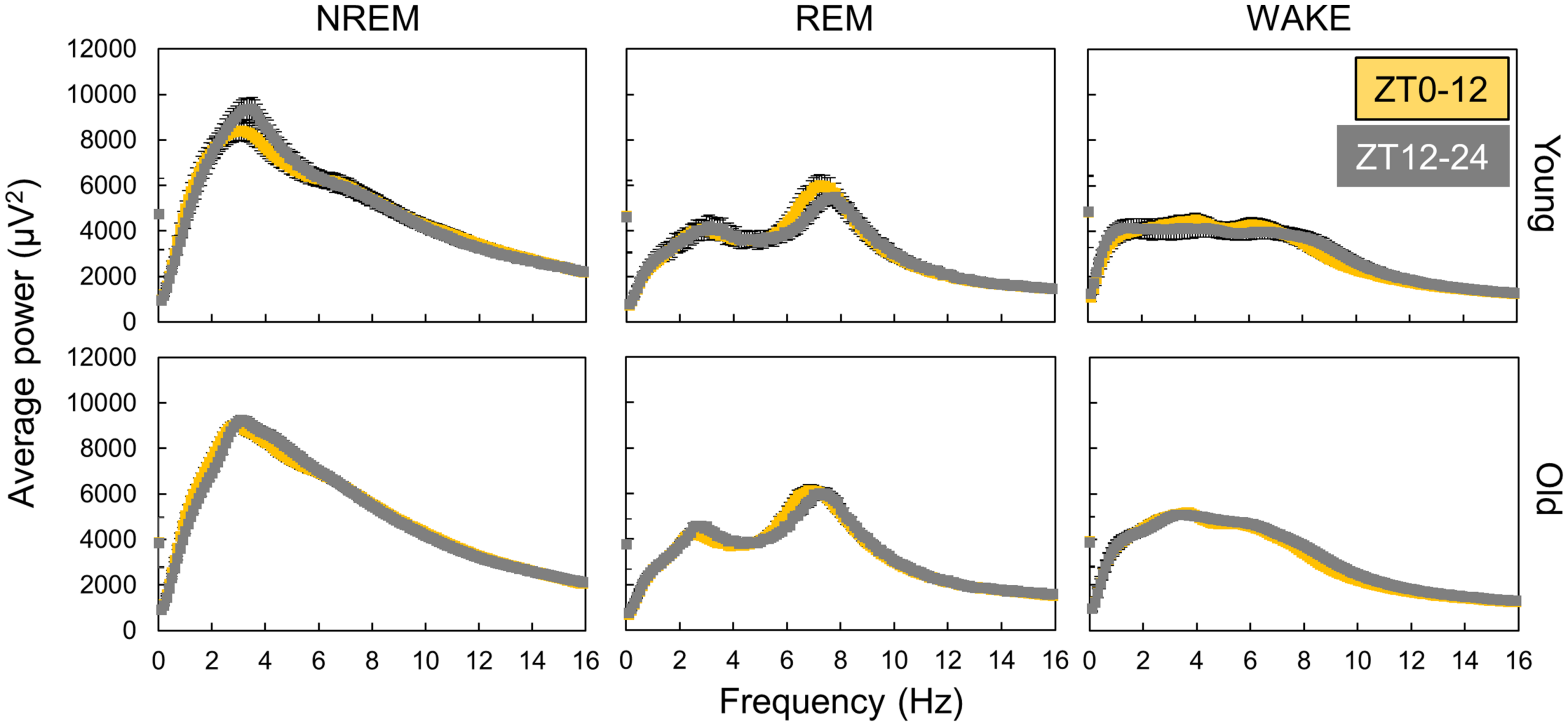
Variation in EEG power spectra during day and night. The points are the average of power spectra during day (ZT0-12) and night (ZT12-24) obtained by Fourier transform from EEG data every 10 s (*n* = 13 in young mice and 20 in old mice in LD). The interval of frequency is 0.1 Hz. Error bars indicate standard error. Yellow and gray areas indicate light and dark conditions, respectively.

We utilized Fourier transformation to calculate EEG power spectra from data collected every 10 s and subsequently determined the average power spectra for each sleep–wake stage. The spectra varied between the light and dark phases for each sleep–wake stage (Figure 3), indicating that sleep quality was time-dependent. However, aged mice exhibited less pronounced variations in the EEG power spectra during NREM sleep between the light and dark periods than young mice. Similar fluctuations in the spectra were also observed between the subjective day (ZT0-12) and night (ZT12-24) under DD conditions (Supplementary Figure S5), implying the involvement of the circadian clock.

**Figure 4:**
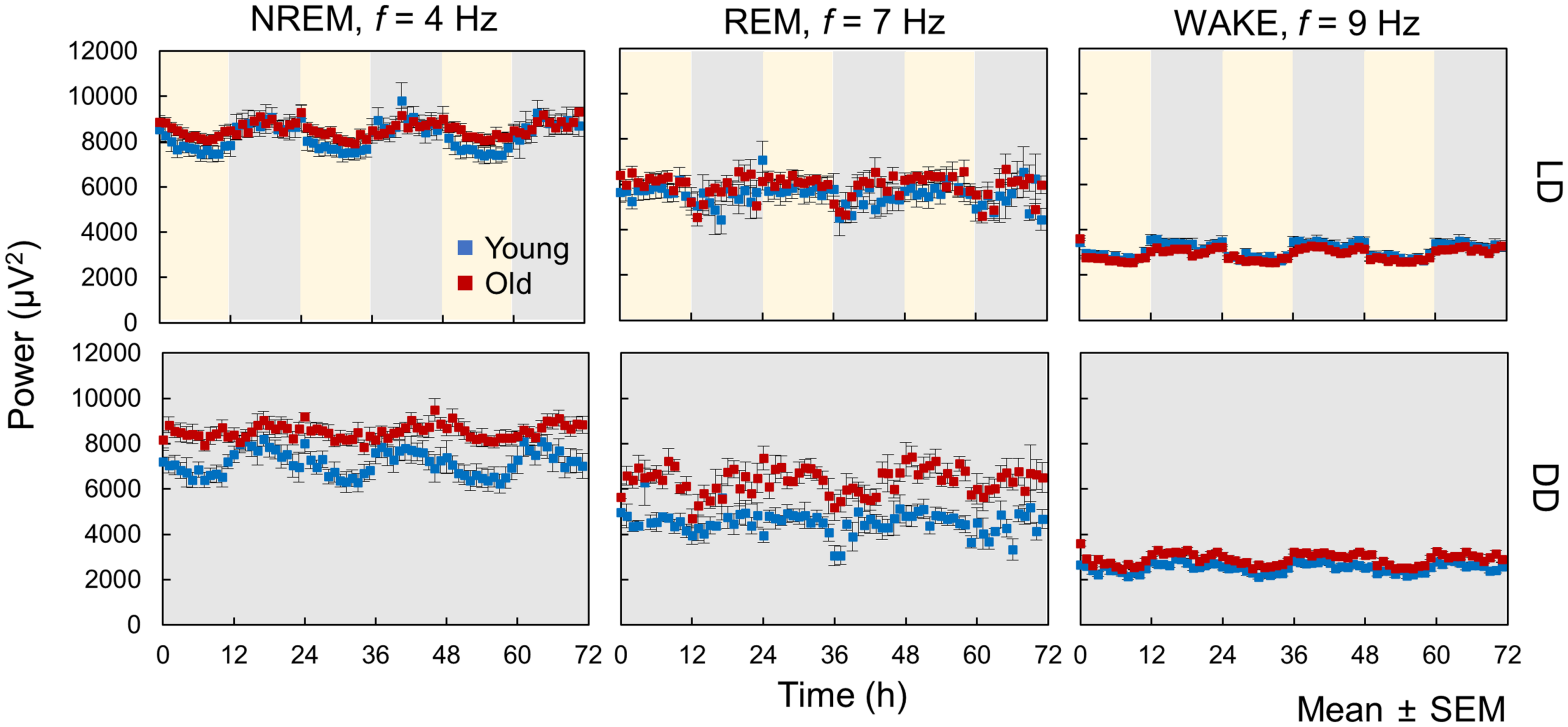
Circadian oscillation in EEG power. These data represent hourly averages of EEG power at 4 Hz for NREM, 7 Hz for REM, and 9 Hz for WAKE (*n* = 13 in young mice and 20 in old mice in LD, 8 in young mice and 10 in old mice in DD conditions). Error bars indicate standard error. Blue and red dots represent values for young and old mice, respectively. Yellow and gray areas indicate light and dark conditions, respectively.

We calculated the hourly averaged EEG power and presented selected examples of EEG power rhythms at a specific frequency, where the changes in EEG power were large between day and night (Figure 4). Diurnal fluctuations in EEG power were evident across all sleep–wake stages in both the LD and DD conditions. The delta wave, generally regarded as a < 4 Hz wave in NREM sleep that reflects drowsiness, exhibited reduced variability in aged mice during both LD and DD (Figure 4). Moreover, the rhythm amplitudes of EEG power at 9 Hz during wakefulness in old mice were smaller than those of young mice in LD but not in DD, suggesting that light input may influence EEG power spectra and that this effect can be attenuated by aging.

**Figure 5:**
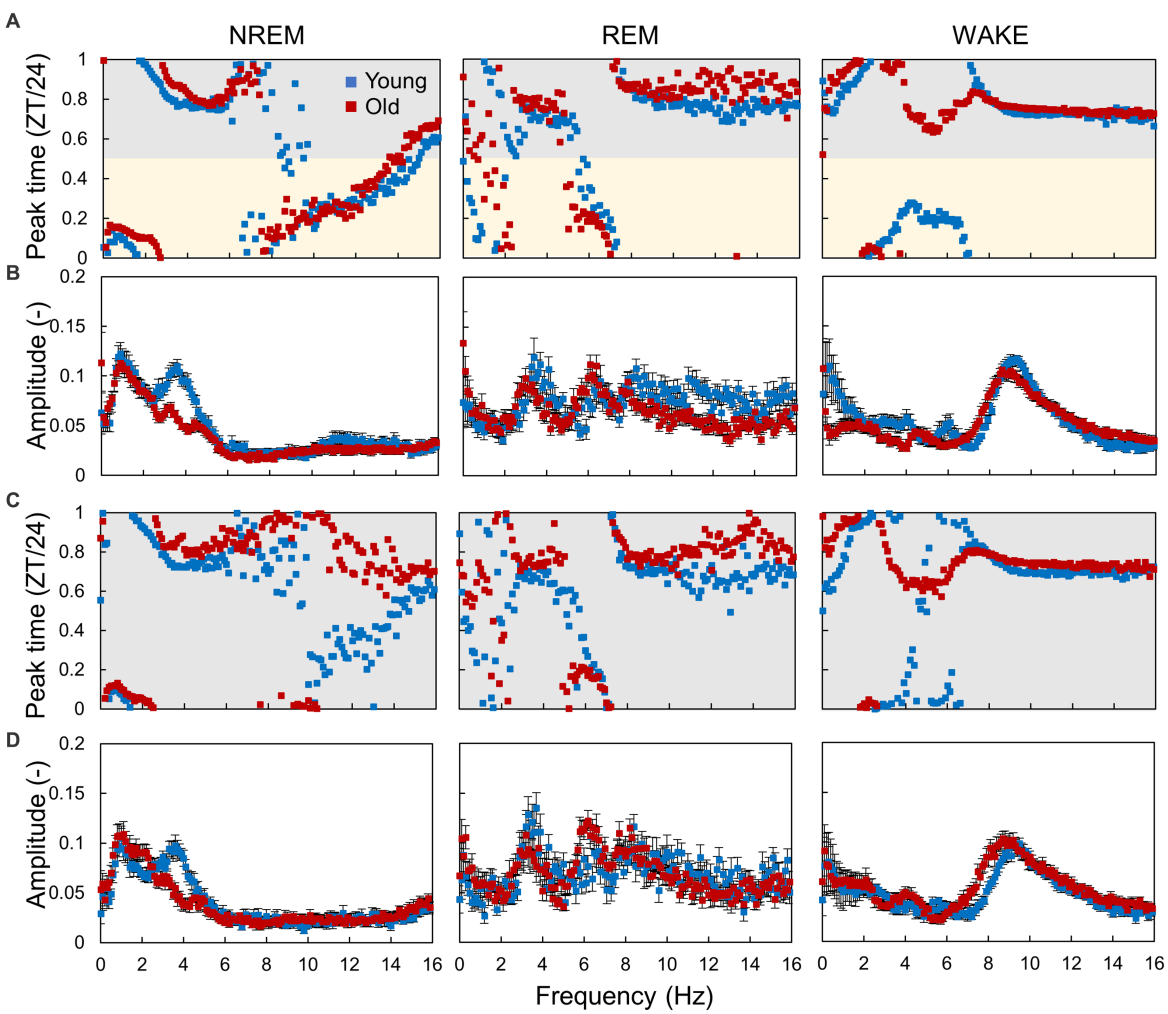
Circadian rhythm components in EEG power. **(A,B)** Peak time **(A)** and amplitude **(B)** of the EEG power rhythm at each frequency obtained from the hourly EEG power spectra in LD conditions. **(C,D)** Peak time **(C)** and amplitude **(D)** of the EEG power rhythm in DD conditions. Error bars indicate standard error. Blue and red dots represent values for young and old mice, respectively. Yellow and gray areas indicate light and dark conditions, respectively. The interval of each frequency is 0.1 Hz. The results of the comparison of young and old mice by Mardia–Watson–Wheeler test for peak time and *t*-test for amplitude at each time point are shown in Supplementary Figure S6.

To clarify the changes in circadian rhythm components in EEG with age, we calculated the amplitude and peak time of EEG power and EMG amplitude rhythms at each sleep–wake stage and frequency (Figure 5; Supplementary Figure S6). In the LD condition, EEG power at different frequencies peaked at different environmental times, in which the peak time continuously fluctuated according to the frequency (Figure 5A; Supplementary Figure S7A). The peak times of the specific frequencies also varied among sleep–wake stages. Peak times of EEG power rhythms tended to be distributed in the middle of the light or dark period (ZT = 6 or 18). However, some frequencies, such as 2 Hz in NREM, showed a peak around ZT0, suggesting that the rhythm of EEG power was not a result of the response to the light–dark transition but was regulated by an intrinsic signal from the circadian clock. The amplitude of the EEG power rhythms varied among frequencies at each sleep–wake stage (Figure 5B; Supplementary Figure S6B). Compared with that in young mice, the peak time in old mice was delayed at most frequencies in NREM sleep and significantly shifted around 5 Hz in wakefulness. Interestingly, the peak time calculated by the amount of wakefulness showed no significant difference between the two groups (Supplementary Figure S3), indicating that the circadian sleep phase cannot be precisely evaluated only by the duration of sleep–wake stages. In contrast, the amplitude of EEG power rhythms at 4 Hz in NREM was significantly decreased in old mice (Figure 5B), as previously shown (Figure 4). In DD, the EEG power rhythms also exhibited amplitudes and peak times similar to those in the LD condition (Figures 5C,D). These results indicate that the rhythms in EEG power were autonomous and independent of light signals, in other words, regulated by the circadian clock. We also observed that the effects of aging were distinct at different sleep–wake stages.

### 3.3. Estimation of circadian time in aged mice

The evaluation of circadian rhythms typically involves determining the amplitude and phase (peak time) of the rhythm per cycle using methods such as cosine fitting. However, changes in circadian rhythms can be time-dependent, and a method that obtains a single phase and amplitude from the rhythm of multiple cycles may not capture the time-dependent changes in circadian rhythm. Notably, in old mice, sleep amounts significantly increased during only the night period (Figure 1), showing time-specific alternation. In contrast, recent studies have suggested a method to estimate the subjective time in individuals from RNA-seq data obtained at a single time point (Hesse et al., 2020). The molecular timetable method, a fundamental time estimation method that employs mRNA expression data, estimates subjective time by identifying which rhythmic genes are at peak or trough expression (Ueda et al., 2004). For example, if the Period genes that exhibit peak expression around CT12 are at their highest, the samples’ subjective time is estimated as CT12. As depicted in Figure 4, the EEG power spectra showed different peak times depending on the frequency. In other words, each spectral distribution is presumed to possess a unique shape at each circadian time, enabling the estimation of mice’s subjective time through spectral distributions.

In this study, we developed a circadian time estimation model based on the EEG power spectrum. To build the model, we used several fundamental machine learning algorithms, such as linear regression, k-nearest neighbor method, ridge regression, support vector machine, and random forest algorithm, to identify the most effective method for predicting the phase. The inputs were set to hourly normalized power spectra in NREM sleep, REM sleep, or wakefulness, hourly normalized power spectra without distinguishing sleep–wake stages, and all sets of spectra in each sleep–wake stage. We used normalized power spectra to reduce experimental errors in the EEG power, and the circadian rhythm component seen in Section 3.2 was also observed in the normalized power spectra (Supplementary Figures S8–S10). The outputs were set to the environmental time.

**Figure 6:**
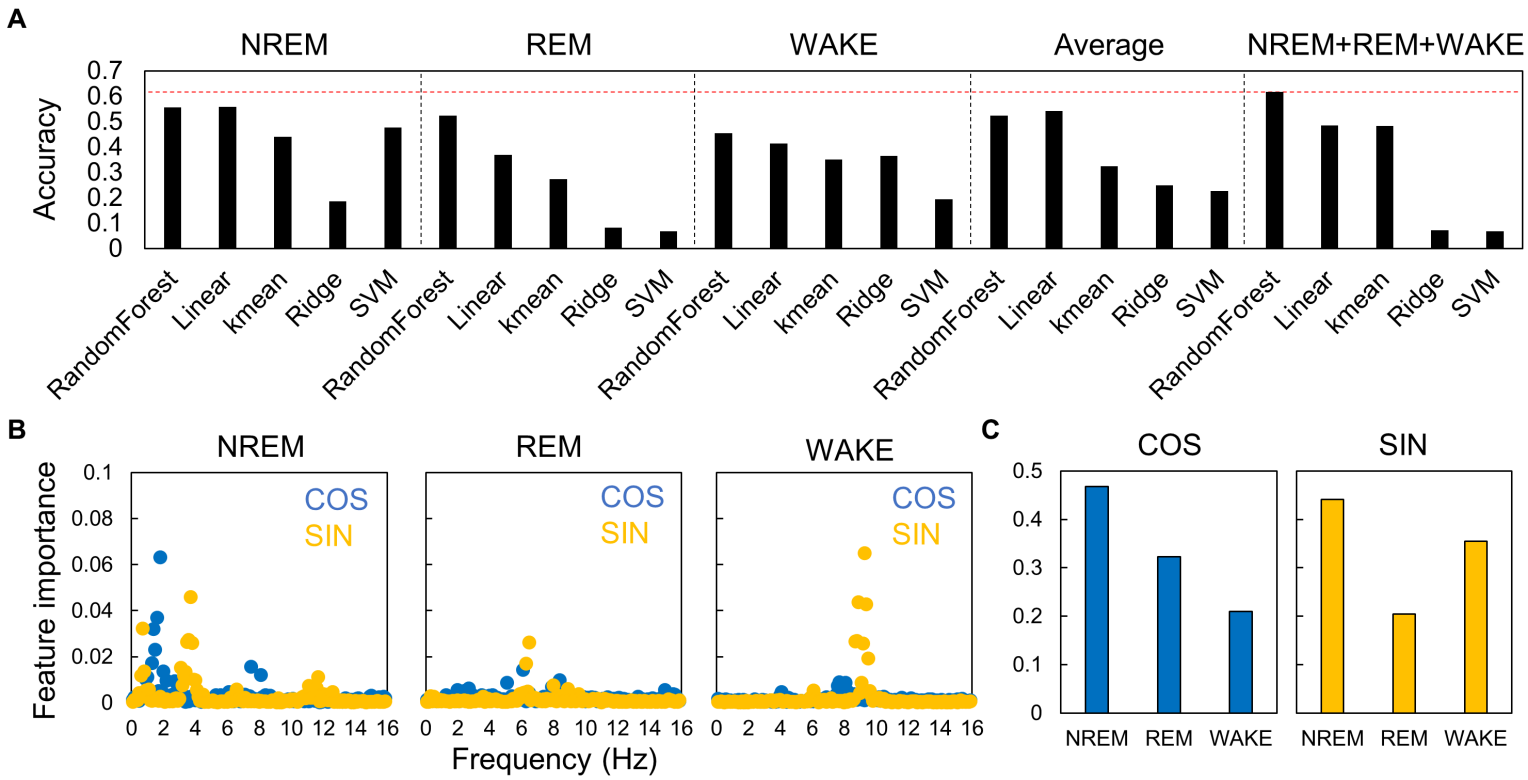
Accuracy of estimation of circadian phase using EEG power spectra in different algorithms. **(A)** Accuracy of the models constructed by each algorithm. The accuracies are shown for each sleep–wake stage, for hourly spectral averages that do not distinguish between sleep–wake stages, and for spectra of all stages. The red dotted line indicates the highest value. Each value is the average of five trials. SVM, support vector machine. **(B)** Importance of each frequency at each sleep–wake stage for time estimation. Blue dots (COS) represent the importance of morning/evening discrimination, and yellow points (SIN) represent the importance of day/night discrimination. **(C)** Importance of each sleep–wake stage for time estimation.

First, to assess the accuracy of each model, we conducted leave-one-out cross-validation using data from young mice in the LD condition (Figure 6A). Among the various machine learning algorithms, the random forest algorithm showed superior performance for all input types. The best performance was achieved when the sets of EEG power spectra and information on the sleep–wake stages were used. Feature importance analysis revealed that frequencies with larger circadian amplitudes, such as 4 Hz in NREM sleep (Figure 5), showed higher importance (Figure 6B). The estimation of circadian time can be broadly categorized into discriminating between day and night (SIN) and morning and evening (COS), and the EEG spectrum in NREM was the most important factor for both discriminations (Figure 6C). This indicates that the shape of the EEG spectrum in NREM changes more prominently depending on the phase of the circadian rhythm than in the other sleep–wake stages.

**Figure 7:**
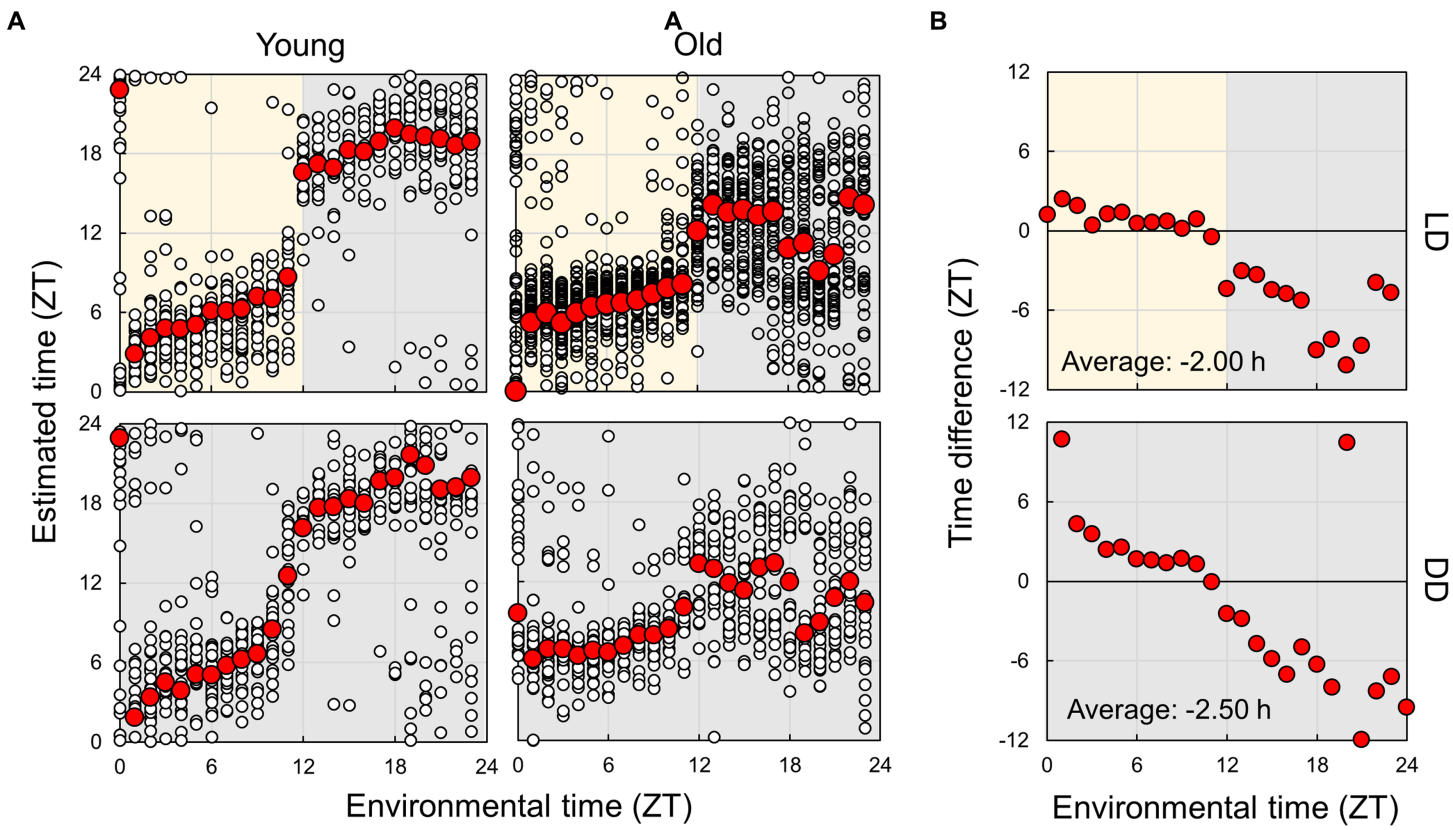
Estimation of subjective circadian phase based on EEG power spectra. **(A)** Estimated subjective time of mice in each condition using the model built based on the EEG of young mice in LD conditions. Blank circles represent the individual data, and red points represent the mean value of each time. **(B)** Difference in estimated time between young and old mice. The values shown in the figures are averages of time differences. Yellow and gray areas indicate light and dark conditions, respectively. The results of the comparison of young and old mice by Mardia–Watson–Wheeler test at each time point are shown in Supplementary Figure S8.

Next, we used an estimation model constructed using the random forest algorithm, utilizing data from young mice under LD conditions, to forecast subject times (Figure 7A). Initially, we compared the actual time and estimated time by employing data from young mice in LD and DD conditions. The estimated times were close to the actual times, whereas there was a substantial shift in the light–dark transition (ZT11-12), which was smaller in DD than in LD. Under the DD condition, where light does not cause an effect, the EEG spectrum may gradually alter in a time-dependent manner around ZT12. We further carried out the estimation by employing the model established from young mice in the DD condition, yielding similar results (Supplementary Figure S11). These results indicate that the estimation model created from data from young mice can be used to assess the spontaneous circadian rhythm of mice.

**Figure 8:**
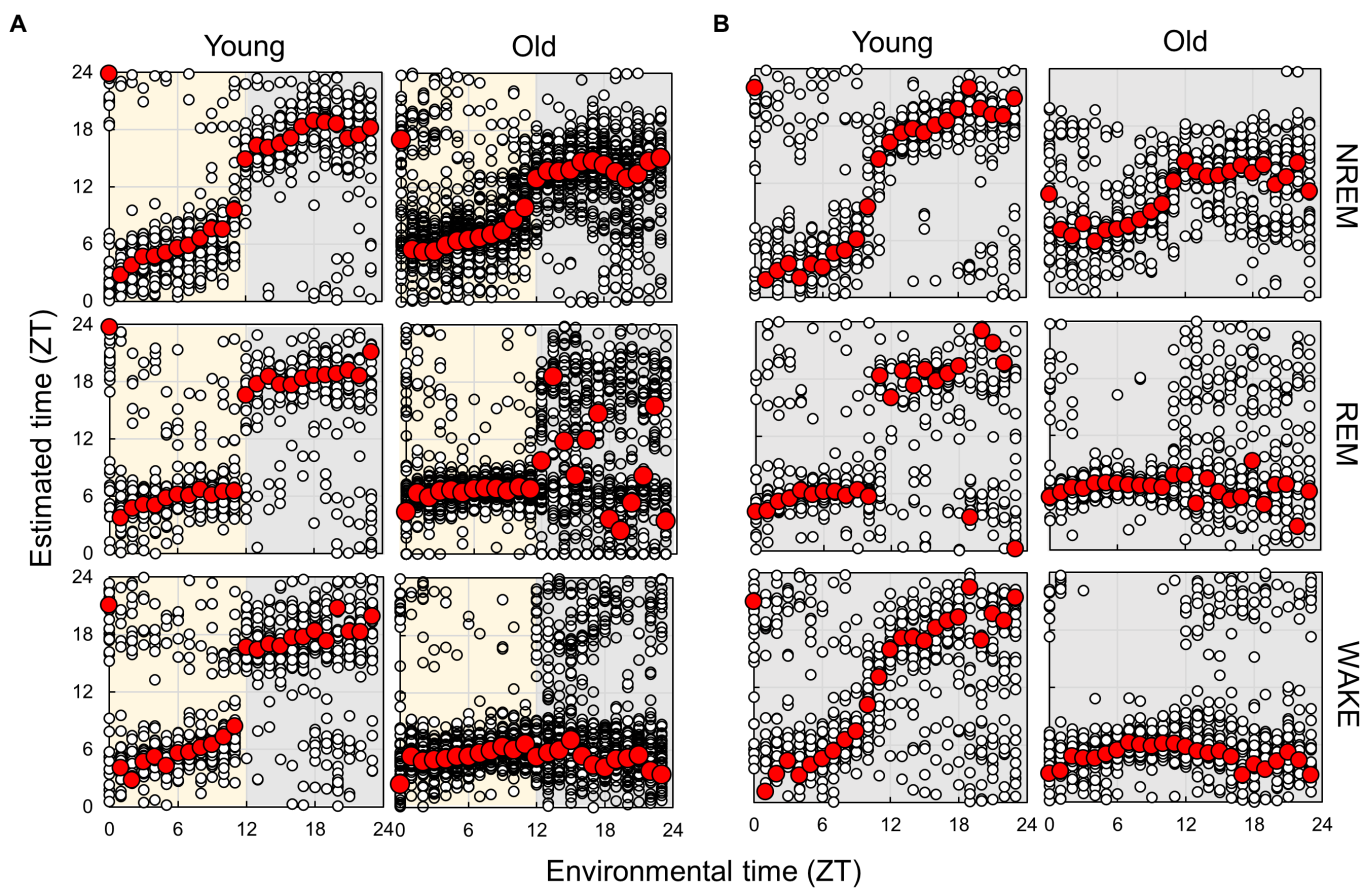
Subjective circadian phase in each sleep–wake stage in LD **(A)** and DD **(B)** conditions. Blank circles represent the individual data, and red points represent the mean value of each time. The time difference between young and old mice and the results of the comparison of young and old mice by Mardia–Watson–Wheeler test at each time point are shown in Supplementary Figures S13, S14.

Finally, we compared the subject time between young and old mice (Figure 7; Supplementary Figure S12). Under LD conditions, although a small effect was observed during the light period, the old mice showed a large delay in circadian time during the night period. The results obtained under the DD condition also showed a phase delay during the subjective night period but a small advancement during the light period. These results indicate that the circadian phase underwent a delay with aging; however, this change was dependent on the environmental time. To check the accuracy of these results, we also trained the model on the dataset of old mice in LD and predict the circadian phase in young and old mice (Supplementary Figure S13). The results showed a similar delay of the estimated time in old mice in the subjective dark period in DD, and an overall time delay in LD. This confirmed that the circadian rhythm of old mice tend to be delayed. However, the phase delay in the dark period was modest compared to the results of the model trained using the dataset of young mice. We also estimated the subject time based on the spectrum of each sleep–wake stage. We observed that the phase in old mice was delayed at night during NREM sleep, although the circadian times were strongly disturbed during REM and wakefulness at night (Figure 8; Supplementary Figure S14). In particular, the circadian fluctuation in the amount of REM sleep was not largely affected (Figure 2); nevertheless, the phase estimated by the spectral power was disturbed during REM sleep (Figure 8).

## 4. Discussion

In this study, we analyzed the sleep–wake patterns of young and aged mice and extracted the circadian rhythm component of each EEG power spectrum to simultaneously assess sleep and circadian rhythms modulated by aging. First, we confirmed the reduction in the amplitude of rhythms in sleep–wake patterns with aging owing to a decrease in wakefulness during the dark period (Figure 2; Wimmer et al., 2013; Panagiotou et al., 2017; McKillop et al., 2018). Subsequently, we extracted the circadian rhythm components in the EEG power spectra at each stage. The presence of spontaneous rhythms in EEG power spectra, even under constant dark conditions (Figures 3–5), suggests regulation by the internal clock. The peak time and amplitude of the EEG power rhythms varied according to the frequencies and sleep–wake stages. Notably, in aged mice, the amplitude of the circadian rhythm in the EEG tended to be attenuated, particularly during NREM sleep and wakefulness. Furthermore, we employed machine learning to evaluate the modulation of circadian rhythms in aged mice with a high temporal resolution (Figure 7). We showed that the phase of circadian rhythm was delayed in aged mice only during the dark period, and the delay was more obvious during REM sleep and wakefulness than in NREM sleep. These results confirm that the attenuation and phase delay of circadian rhythms with aging occurred at the EEG level and that the effects of aging on circadian rhythm and sleep–wake behavior depend on sleep–wake stages (wakefulness, NREM or REM) and time of day (circadian phase), respectively.

We extracted circadian rhythm components from the EEG power spectrum. The results showed different circadian rhythms among sleep–wake stages. Previous studies also showed that the delta power (0.5–4 Hz) rhythm of NREM has a peak around ZT0 (Wisor et al., 2008; Wimmer et al., 2013), and the 5–7 Hz rhythm of REM and WAKE has a peak around ZT6 (Yasenkov and Deboer, 2010). These results are consistent with our results (Figure 5). In aged mice, the amplitude of the EEG power rhythm was attenuated, especially at 4 Hz (delta wave), during NREM. Delta power is an indicator of sleepiness or sleep quality because the lack of sleep, such as sleep deprivation, strengthens it (Åkerstedt and Gillberg, 1990; Wisor et al., 2008). Thus, it is predicted to be rhythmic, lagging behind the sleep–wake pattern, as shown in our results (Figure 5). However, although delta wave usually refers to between 0.5–4 Hz wave, the peak time of the EEG power rhythm and the effect of aging vary even between them (Figure 5). 0.5 Hz wave showed the peak during the light phase and was not affected by aging. On the other hand, the rhythm of 4 Hz wave power peaks in the middle of the night and was significantly attenuated by aging. Therefore, it is likely that these delta powers are largely influenced by sleep pressure, but also affected by other factors such as the circadian clock. The amount of sleep in the old mice increased at night, and the amplitude of the rhythm of the sleep–wake pattern decreased (Figure 2). These results indicate an association between circadian rhythm attenuation and aging and sleep disorders in the elderly. Moreover, disturbances in circadian rhythm in old mice also appeared at night, which is the active period for these animals (Figure 8). Although it remains unknown whether these changes are caused by changes in either circadian rhythm or sleep, the fact that age-associated changes in circadian rhythm and sleep–wake behavior are time-dependent suggests the need for time-dependent treatment of sleep and circadian rhythm disorders.

Although many studies have analyzed EEG for sleep analysis, few studies have focused on the phase of the circadian rhythm. In general, manual EEG analysis is time-consuming and can create bias by the analyst, making it difficult to evaluate circadian rhythms using the same criteria. Thus, we used the neural network model developed in a previous study (Tezuka et al., 2021) for sleep–wake stage scoring. One intent of this analysis was to demonstrate that circadian rhythms can be analyzed using large-scale EEG data. By combining automated sleep–wake stage scoring with circadian time prediction, it is possible to analyze circadian rhythms for large-scale data from various EEG databases without any bias. Notably, because the estimation method does not require long-term (over 1 d) recording of EEG to output the circadian phase, short-term EEG data, which were not intended to examine circadian rhythms, can be subjected to re-analysis using our method. We utilized this model in the current study because one of its features is the use of environmental time as one of its inputs, which reduces the time-dependent sleep scoring errors. In addition, because the model is capable of real-time analysis of sleep–wake stages, real-time circadian rhythm phase analysis would also be possible using our methods. Newer models have been proposed for machine learning EEG analysis; therefore, the accuracy of circadian rhythm phase estimation may also be improved using the latest models (Craik et al., 2019; Phan and Mikkelsen, 2022; Koyanagi et al., 2023). We propose that automated and spontaneous evaluation of sleep and circadian rhythms can be useful for high-throughput mutant screening, in which sleep and circadian rhythms are usually evaluated by separate screenings.

We estimated the subjective circadian time of mice using machine learning, and the estimated times were proportional to the environmental time under both LD and DD conditions. This indicates that EEG rhythms at each sleep–wake stage were not merely responses to light–dark transitions but clock-driven spontaneous rhythms and that it is possible to evaluate the phase of circadian rhythms without a long measurement period, as in behavioral analysis. However, even in young control mice, the estimation results were disturbed during the second half of the dark period. This may result from the increase in sleep amount at this time. The method of averaging hourly spectra may not provide sufficient data to evaluate the differences in sleepiness between light and dark periods. Therefore, it is necessary to develop methods that utilize higher-temporal-resolution data for time prediction. For example, previous studies have shown the existence of circadian periodicity in fractal structures in EEG (Croce et al., 2018). A more detailed extraction of circadian rhythm components in EEG will enable the evaluation of circadian rhythm phases with higher accuracy and temporal resolution. Apart from EEG and EMG, mice exhibit various other biological rhythms such as body temperature and endocrine rhythms. Simultaneous measurement of these rhythms with EEG can improve our understanding of circadian rhythm changes associated with aging, and potentially enhance our ability to predict circadian rhythms. The circadian time estimated for the old mice were highly disturbed during the night period (Figures 7, 8). However, the disruption was more modest when based on the old mice dataset (Supplementary Figure S13). It is assumed that when the old mice dataset was used for training, EEG powers that had no or weak rhythms in the old mice were not used for the estimation, leading to a more stable estimated circadian time in the old mice. In other words, the loss of the circadian rhythm component in the old mice might have caused the delay in the estimated time during nighttime in the old mice. Therefore, further development of analysis methods may be necessary to estimate the circadian phase reliably, regardless of the dataset used for training.

As demonstrated by previous studies and the present study, aging has a significant impact on circadian rhythm and sleep. Moreover, circadian rhythm disturbances and sleep disorders also accelerate aging (Kondratov et al., 2006; Sadeghmousavi et al., 2020; Carroll et al., 2021; Acosta-Rodríguez et al., 2022). Therefore, because aging, circadian rhythm, and sleep are mutually influential, it is difficult to evaluate them individually. Furthermore, circadian behavior has been conventionally analyzed using the quantitative fluctuation of behavior (such as counts of wheel-running and amounts of sleep) but not from the qualitative aspect. In the present study, we reconstructed the circadian phases during each sleep–wake stage separately based on spectrum analysis (Figure 8). The results showed that the circadian phases in the aged mice were delayed during NREM sleep, although significantly disrupted during REM sleep and wakefulness, indicating that aging affects circadian rhythms during each stage; however, these mechanisms may be independent. The present study indicates that analysis of circadian rhythms using EEG is useful to more deeply understand the effect of factors such as aging, which affect both sleep–wake and circadian rhythms.

## Data availability statement

The original contributions presented in the study are included in the article/Supplementary material, further inquiries can be directed to the corresponding authors.

## Ethics statement

The animal study was reviewed and approved by the Animal Experiment and Use Committee of the University of Tsukuba.

## Author contributions

KM and AH designed this study. YK and YN performed the experiments. KM analyzed the data. KM, YN, TS, and AH wrote the manuscript. All authors discussed the results and implications and commented on the manuscript.

## Funding

This study was partially supported by Grant-in-Aid for JSPS Fellows (no. 22J00270 to KM), for Scientific Research (no. 22K15157 to KM) provided by the Japan Society for the Promotion of Science (JSPS), the Moonshot Research Development Program (no. AGL03337 to AH) provided by Japan Agency for Medical Research and Development (AMED), and Research grant (AH) (The Naito Foundation, Japan).

## Conflict of interest

The authors declare that the research was conducted in the absence of any commercial or financial relationships that could be construed as a potential conflict of interest.

## Publisher’s note

All claims expressed in this article are solely those of the authors and do not necessarily represent those of their affiliated organizations, or those of the publisher, the editors and the reviewers. Any product that may be evaluated in this article, or claim that may be made by its manufacturer, is not guaranteed or endorsed by the publisher.

## Footnotes

^1^ https://github.com/tarotez/sleepstages

^2^ https://lmfit.github.io/lmfit-py

^3^ https://github.com/scikit-learn/scikit-learn
