# Supplementary material for: Analysis of circadian rhythm components in EEG/EMG data of aged mice
Source: Front Neurosci. 2023 May 12;17:1173537. doi: 10.3389/fnins.2023.1173537 (PMC10213445; doi:10.3389/fnins.2023.1173537)
Supplement: Supplementary file 1 [file Data_Sheet_1.docx]

Supplementary Material

# Supplementary Figures

**
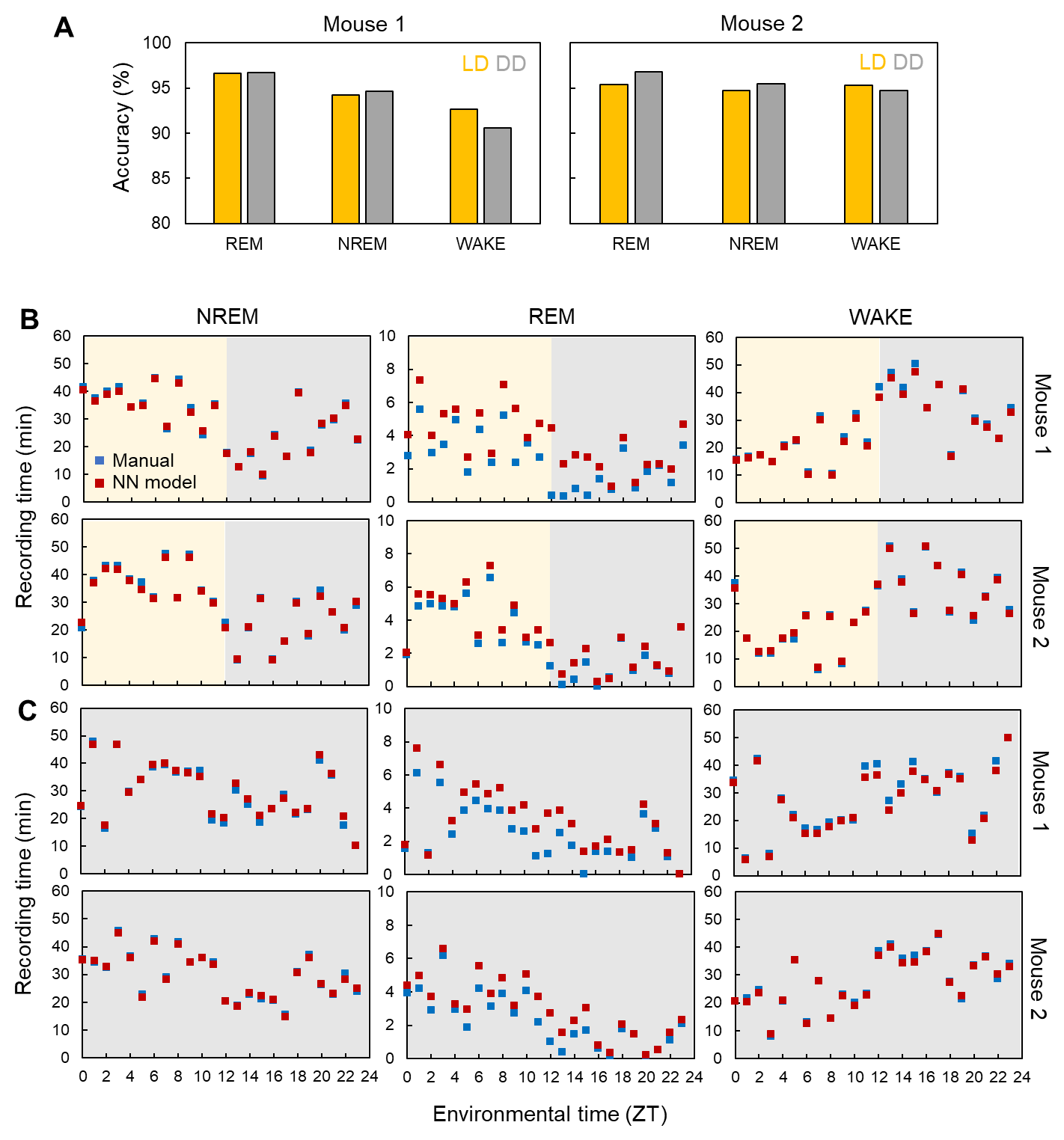
**

**Figure S1.** Validation of sleep-stage scoring using neural network model (NN model). **A** Accuracy of scoring using NN model. Accuracy was calculated as (amount of each sleep-wake stage that match the results of manual scoring in NN model)/(amount of each sleep-wake stage in manual scoring). For calculation of accuracy, sleep-wake stages of two young mice were manually scored. **B** and **C** Amount of each sleep stage in NN model and manual scoring in LD (**B**) and DD (**C**). The yellow areas indicate light condition and the gray areas indicate dark condition.

**
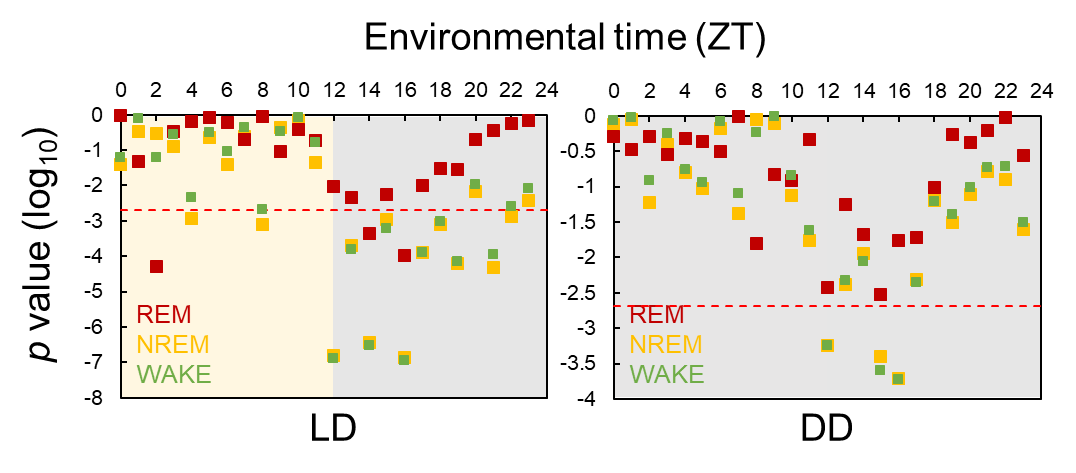
**

**Figure S2.** Results of *t*-tests for comparison of sleep/wake amounts between young and aged mice. Red lines indicate the significant level α=0.05 with Bonferroni correction (=0.05/24). There are significant differences at points below the red line.

**
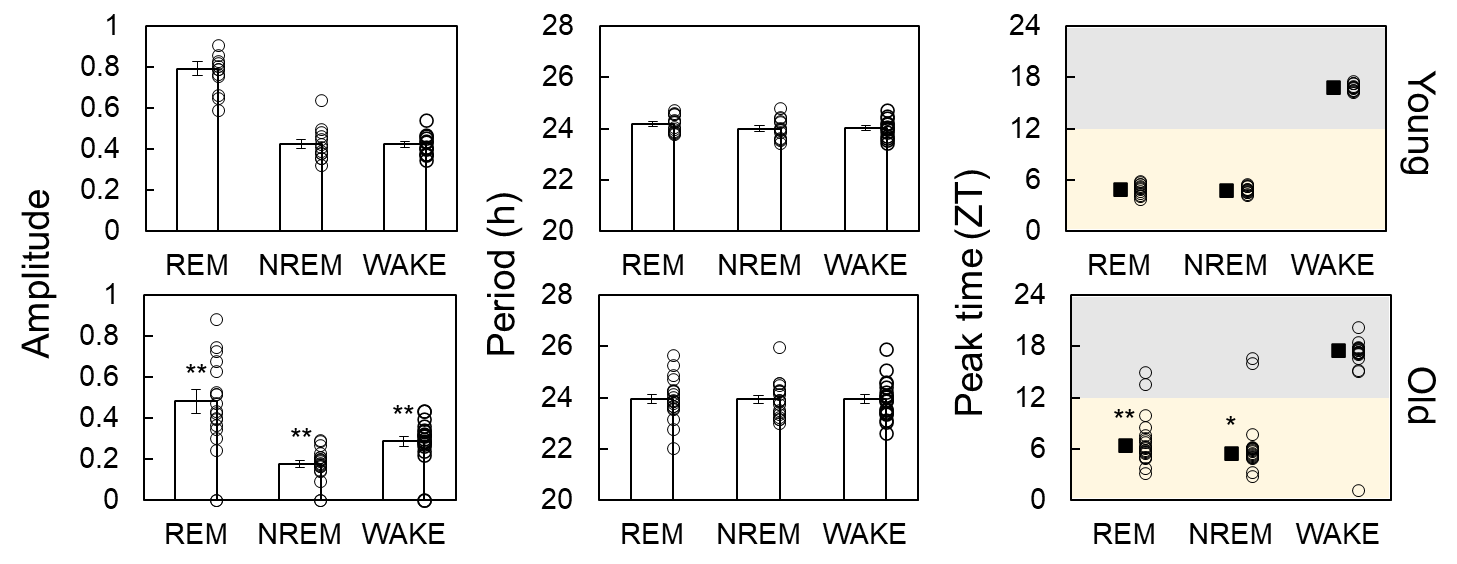
**

**Figure S3.** Amplitude, period and peak time of sleep-wake rhythm in LD condition. Blank circles indicate individuals. Error bars indicate SEM and asterisks indicate the result of significant differences between young and old mice (*: *p*<0.05, **: *p*<0.01, *t*-test in amplitude and period and Mardia-Watson-Wheeler test in peak time).


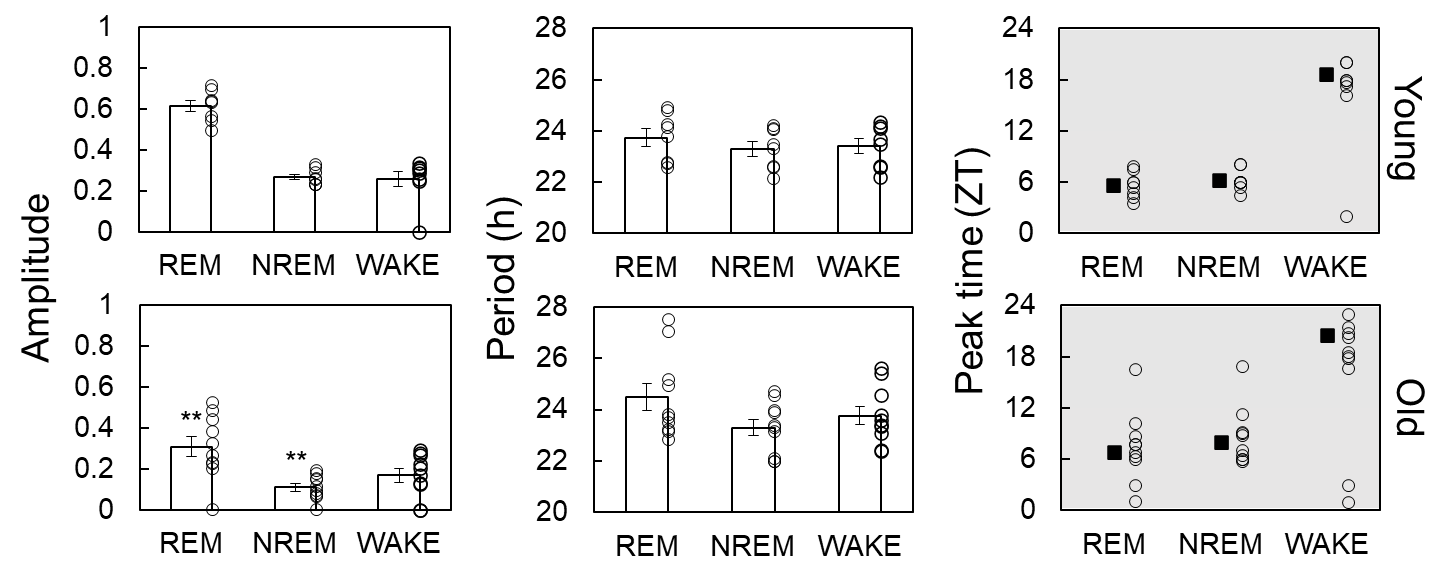


**Figure S4.** Amplitude, period and peak time of sleep-wake rhythm in DD condition. Blank circles indicate individuals. Asterisks indicate the result of significant differences between young and old mice (*: *p*<0.05, **: *p*<0.01, *t*-test in amplitude and period and Mardia-Watson-Wheeler test in peak time).


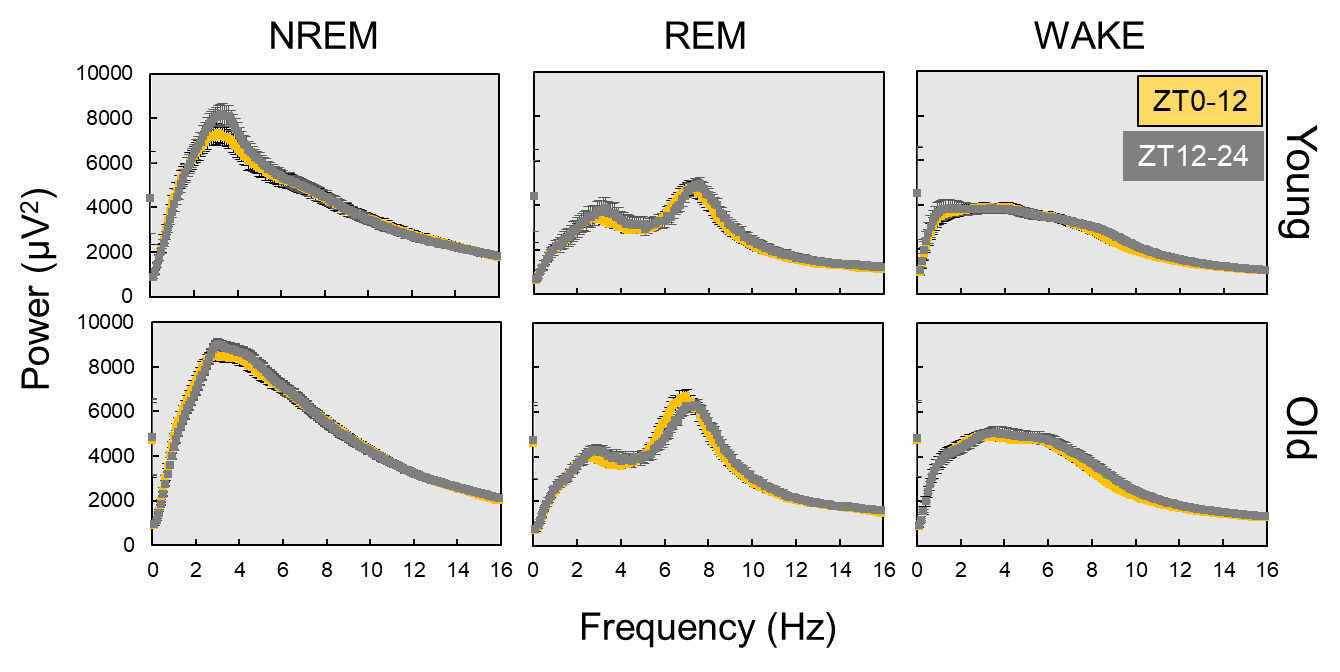


**Figure S5.** Average EEG spectra in ZT0-12 and ZT12-24 in DD condition. Error bars indicate SEM.


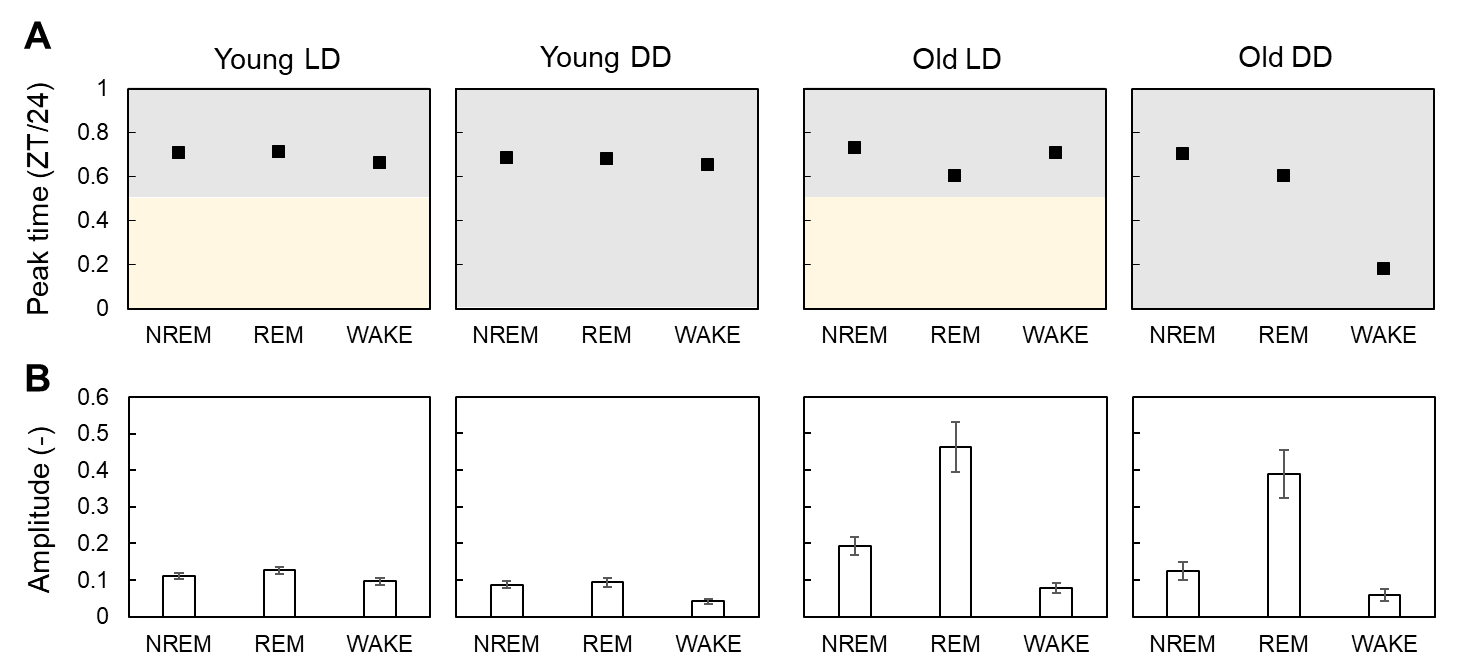


**Figure S6.** Circadian rhythm components of EMG amplitude. **A** Peak time of EMG amplitude rhythms. **B** Amplitude of circadian rhythm of EMG amplitude.


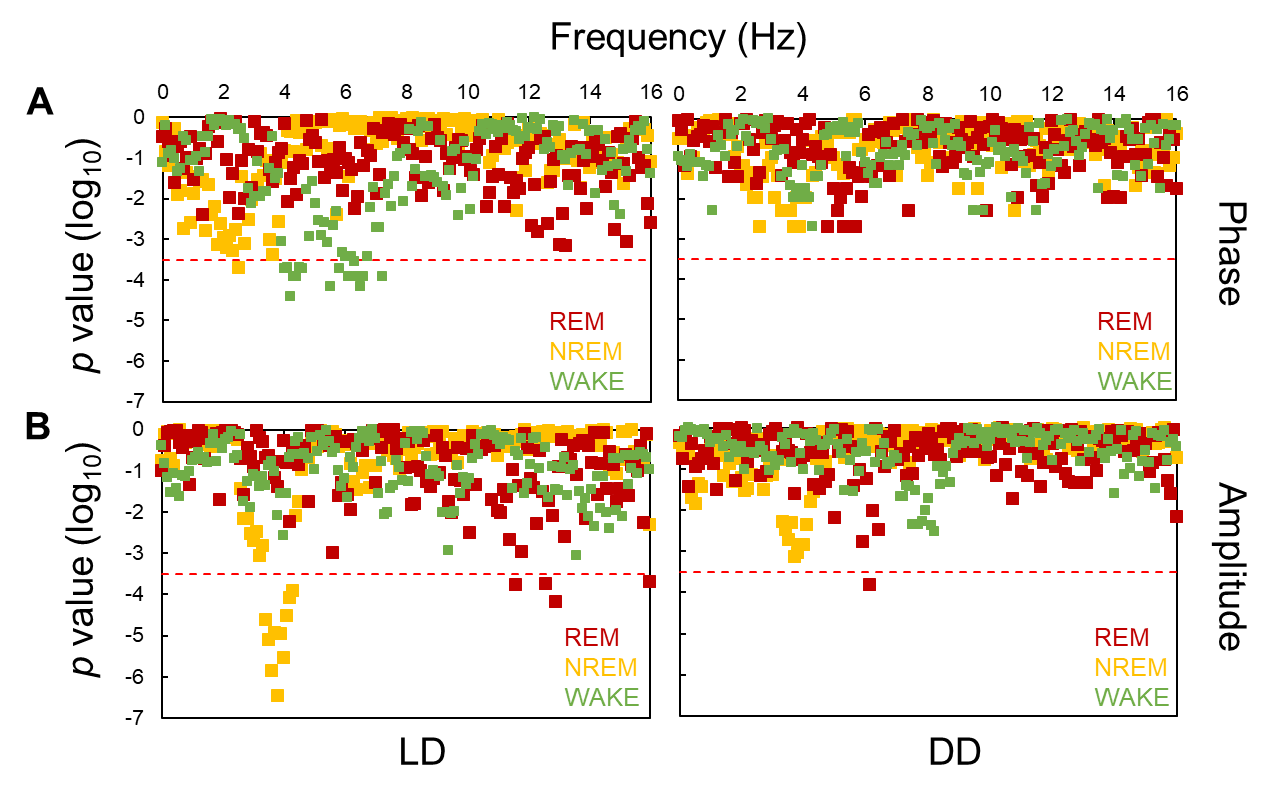


**Figure S7.** *p-*values for comparison of circadian rhythm components between young and aged mice. **A** The results of *t*-test for amplitude of EEG power rhythms. **B** The results of Mardia-Watson-Wheeler test for peal time of EEG power rhythms. The interval of frequency is 0.1 Hz. Red lines indicate the significant level α=0.05 with Bonferroni correction (=0.05/160). There are significant differences at points below the red line.


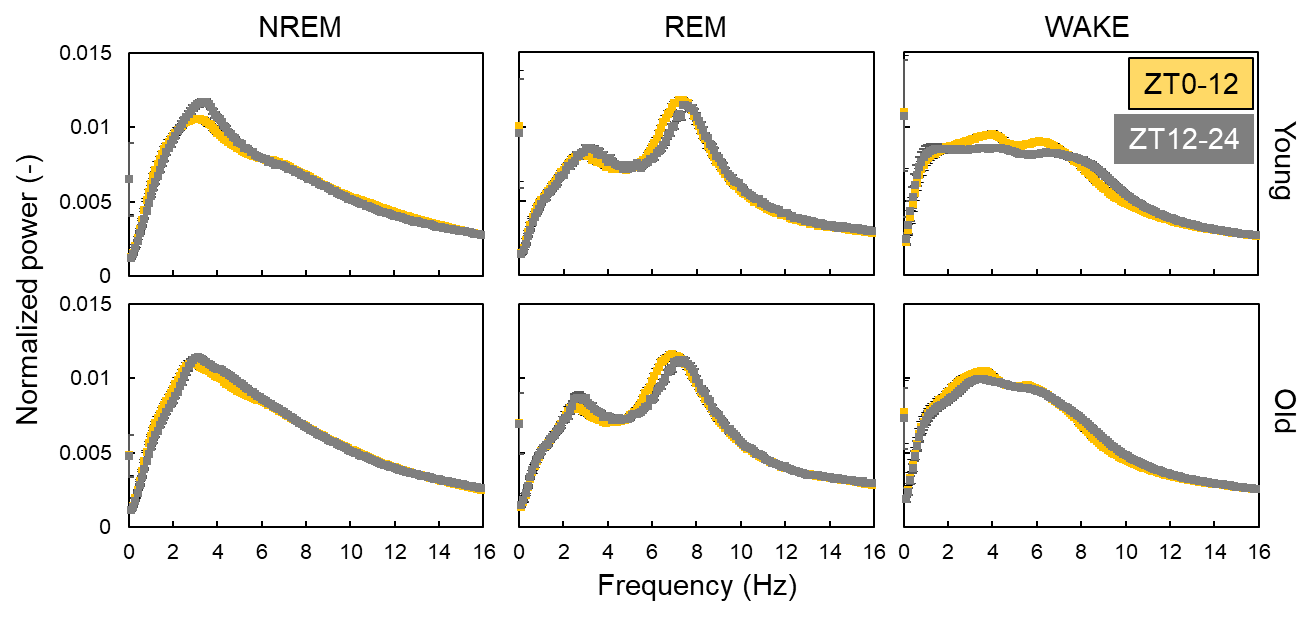


**Figure S8.** Normalized EEG spectra in ZT0-12 and ZT12-24 in LD condition. Error bars indicate SEM.


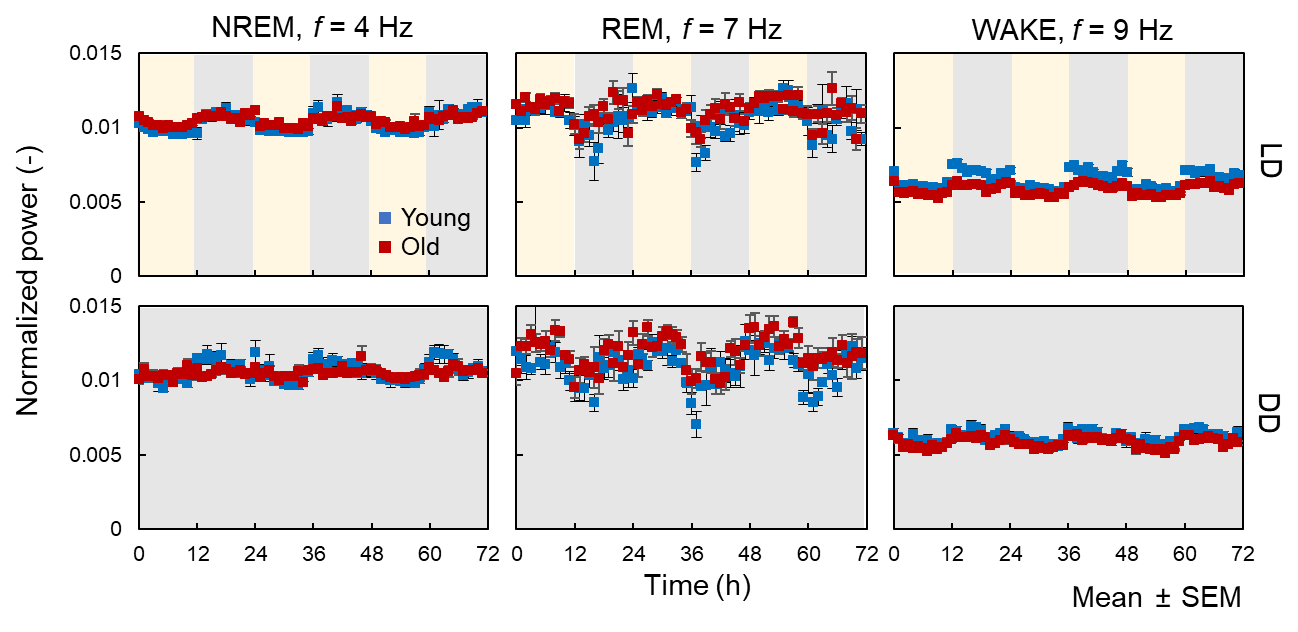


**Figure S9.** Circadian oscillation in normalized EEG power. These data represent hourly averages of EEG power at 4 Hz for NREM, 7 Hz for REM, and 9 Hz for WAKE (n = 13 in young mice and 20 in old mice in LD, 8 in young mice and 10 in old mice in DD conditions). Error bars indicate standard error. Blue and red dots represent values for young and old mice, respectively. Yellow and gray areas indicate light and dark conditions, respectively.


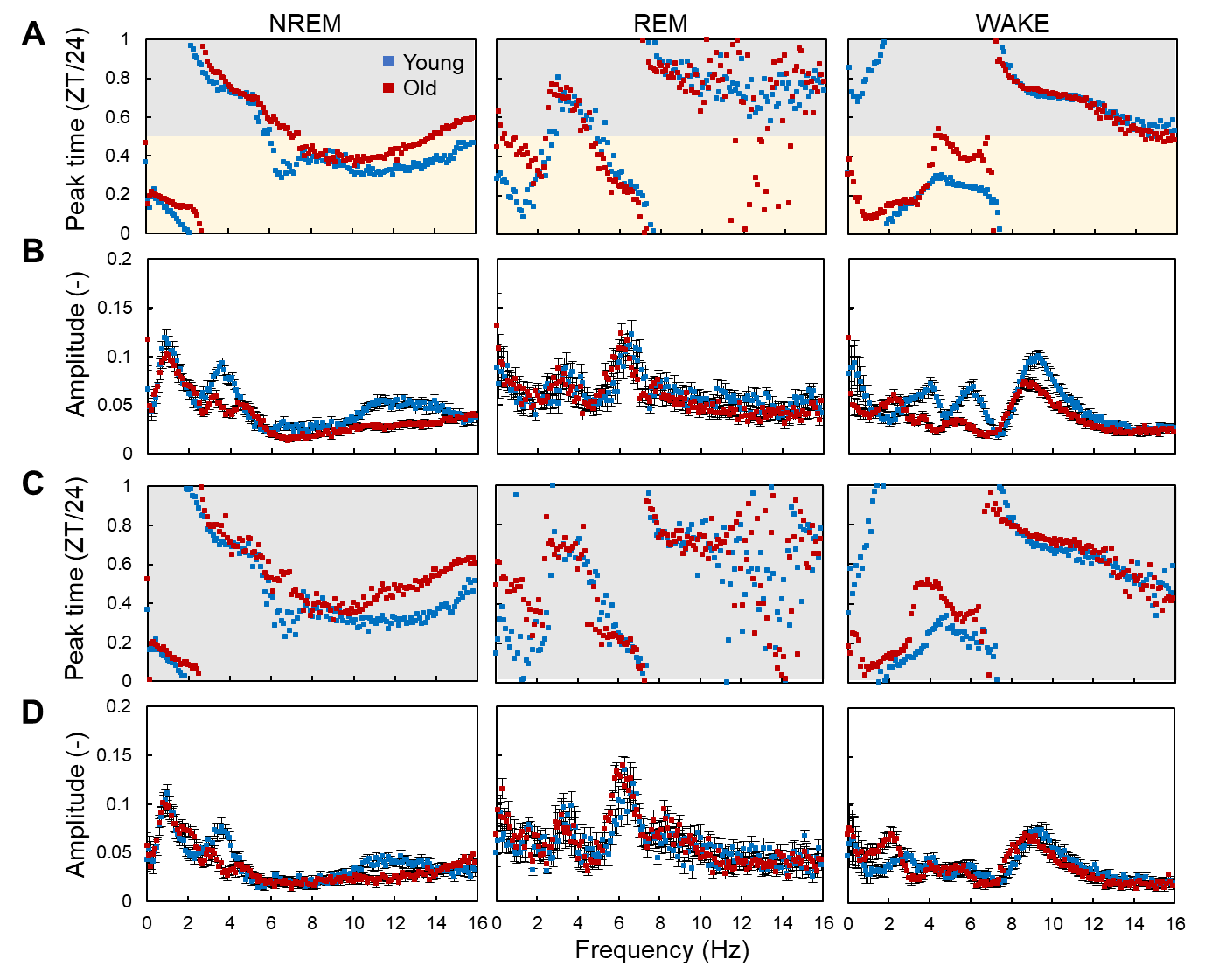


**FIGURE 10** | Circadian rhythm components in normalized EEG power. **A** and **B** Peak time (A) and amplitude (B) of the normalized EEG power rhythm at each frequency obtained from the hourly EEG power spectra in LD conditions. **C** and **D** Peak time (C) and amplitude (D) of the normalized EEG power rhythm in DD conditions. Error bars indicate standard error. Blue and red dots represent values for young and old mice, respectively. Yellow and gray areas in (A) and (C) indicate light and dark conditions, respectively. The interval of each frequency is 0.1 Hz.


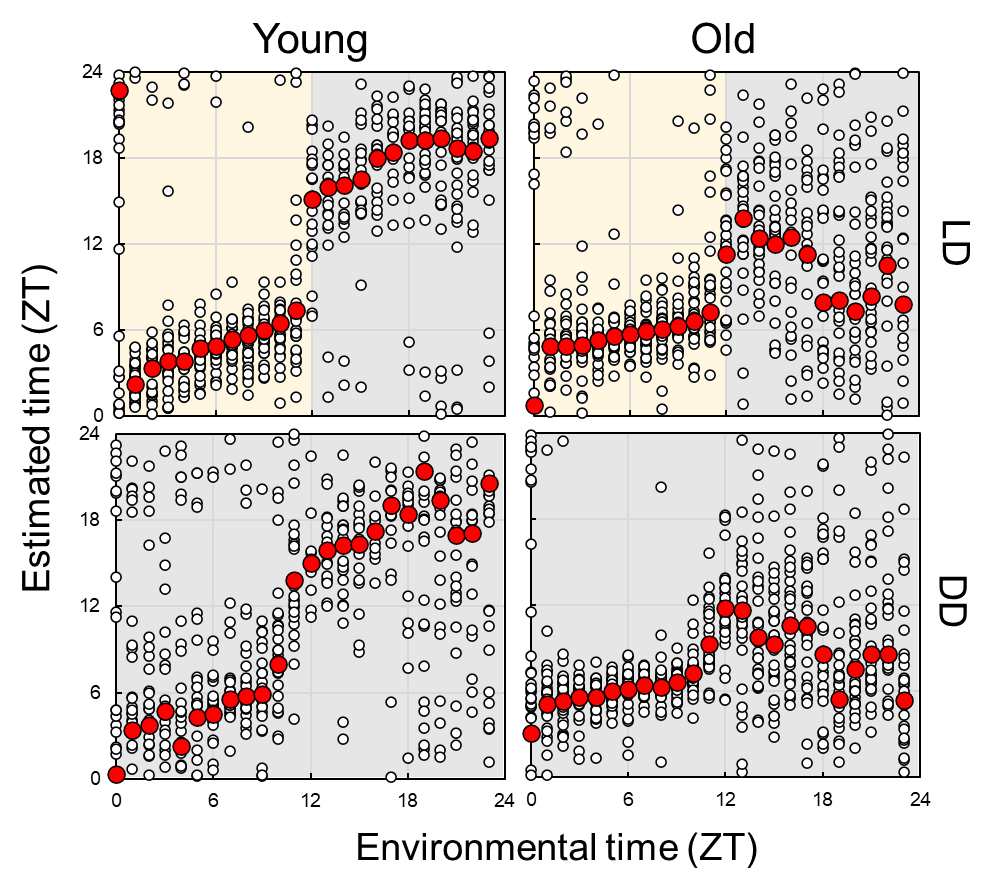


**Figure S11.** Estimation of subjective circadian phase based on the model using EEG spectra of young mice in DD condition. Blank circles represent the individual data, and red points represent the mean value of each time.


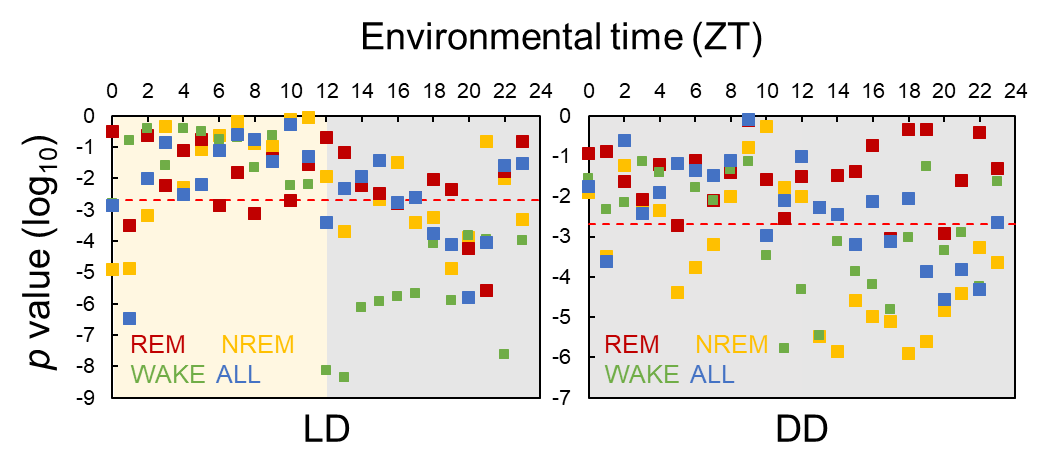


**Figure S12.** Results of Mardia-Watson-Wheeler tests for comparison of estimated circadian time between young and aged mice. Red lines indicate the significant level α=0.05 with Bonferroni correction (=0.05/24). There are significant differences at points below the red line. ALL represents the results of the estimation based on the model using EEG power spectra at all sleep stages, REM, NREM and WAKE.

**
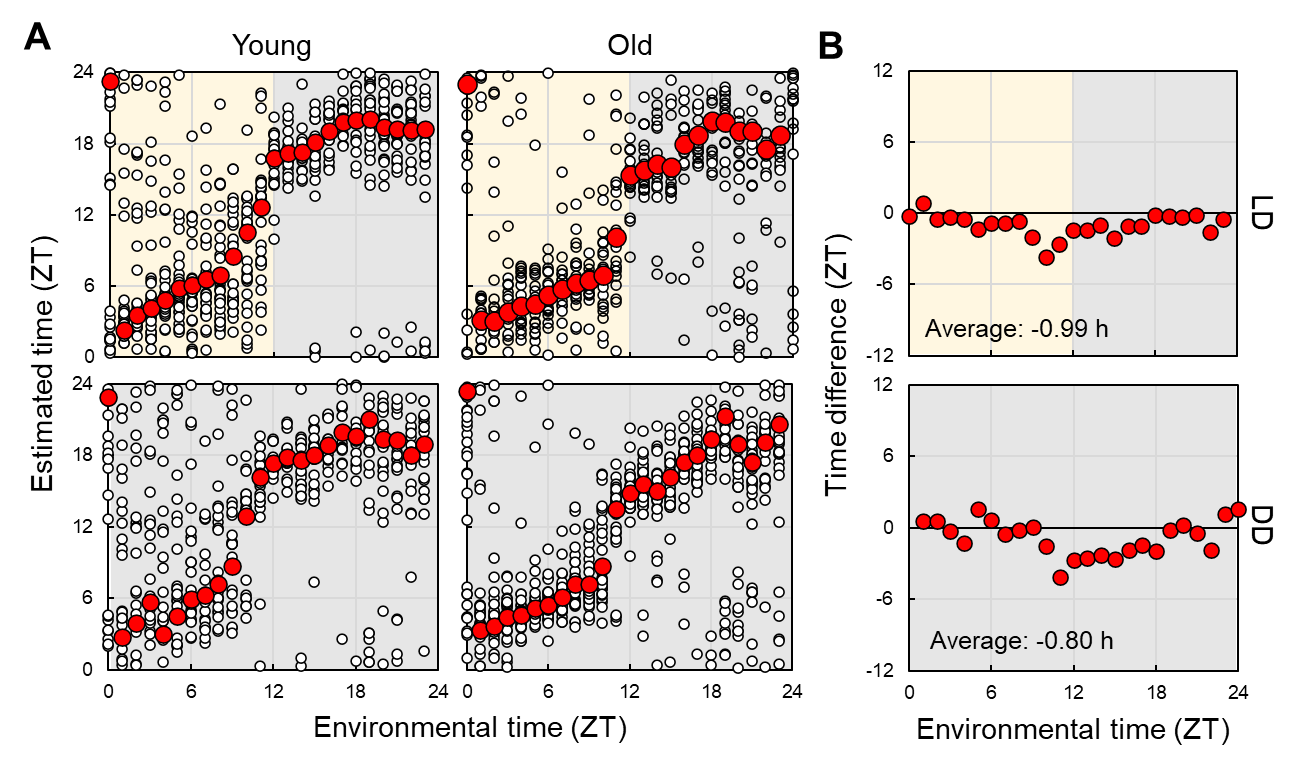
**

**Figure S13.** Estimation of subjective circadian phase based on the model using EEG spectra of old mice in LD condition. Blank circles represent the individual data, and red points represent the mean value of each time.


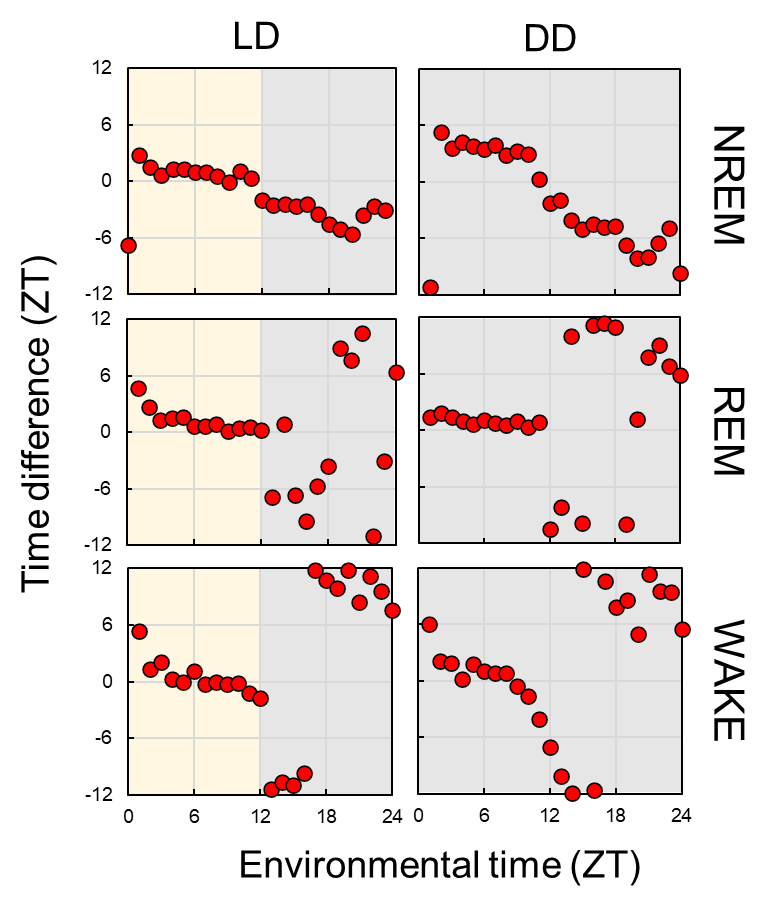


**Figure S14.** The difference in estimated time between young and old mice in each sleep stage. The yellow areas indicate light conditions, and the gray areas indicate dark conditions.
